# Integrative approaches in severe mental illness

**DOI:** 10.1192/j.eurpsy.2026.10258

**Published:** 2026-07-31

**Authors:** R. de Filippis

**Affiliations:** Health Sciences, University Magna Graecia of Catanzaro, Catanzaro, Italy

## Abstract

**Abstract:**

Integrative approaches in severe mental illness (SMI) represent a cornerstone of contemporary psychiatric practice, addressing the multifaceted needs of individuals affected by chronic and disabling mental disorders. This intervention focuses on the practical application of integrative care models that combine biological, psychological, social, and lifestyle-oriented strategies to improve clinical, functional, and recovery-related outcomes.

The presentation highlights how evidence-based pharmacological treatments can be effectively combined with psychosocial interventions, including psychotherapy, family involvement, vocational rehabilitation, and community-based support. Special emphasis is placed on the integration of physical health care, recognising the high burden of cardiometabolic comorbidity and reduced life expectancy among people with SMI. Emerging areas such as digital tools, shared decision-making, and recovery-oriented practices are also discussed as key components of truly integrative care.

Through clinical examples and real-world scenarios, the intervention illustrates common challenges in implementation, such as treatment resistance, comorbidity, fragmentation of services, and inequities in access to care. Strategies to overcome these barriers—particularly relevant for early career psychiatrists—are presented, highlighting the role of multidisciplinary teamwork, continuity of care, and personalised treatment planning across different stages of illness.

Overall, this intervention aims to provide participants with a concise, clinically oriented framework for delivering integrative, person-centred care in SMI, fostering improved outcomes, enhanced quality of life, and sustainable recovery pathways within diverse healthcare settings.

**Disclosure of Interest:**

R. de Filippis Grant / Research support from: Angelini, Johnson & Johnson, Lundbeck, Neuraxpharm, and Rovi, Consultant of: Angelini, Johnson & Johnson, Lundbeck, Neuraxpharm, and Rovi

